# Review on Gelling and Foaming Agents in Food: Mechanism and Applications

**DOI:** 10.1002/fsn3.72437

**Published:** 2026-10-03

**Authors:** Messenbet Geremew Kassa, Desye Alemu Teferi, Neela Satheesh, Aynadis Molla Asemu

**Affiliations:** ^1^ Department of Food Science and Post‐Harvest Technology Injibara University Injibara Ethiopia; ^2^ Department of Food Science and Technology, School of Health Sciences Amity University Mohali Punjab India; ^3^ Faculty of Chemical and Food Engineering, Bahir Dar Institute of Technology Bahir Dar University Bahir Dar Ethiopia

**Keywords:** egg white, foam capacity, food texture, gelling, pectin

## Abstract

Food texture and structural stability are critical determinants of consumer acceptance and overall product quality in the food industry. This review provides a comprehensive synthesis of gelling and foaming mechanisms, focusing on traditional hydrocolloids (pectin and gelatin) alongside emerging plant‐based alternatives, such as aquafaba (chickpea cooking water). For gelling systems, we examine the molecular networks driving matrix formation, highlighting how processing variables and natural source variability complicate texture control in clean‐label formulations. For foaming systems, the mechanisms of surface tension reduction, gas incorporation, and interfacial stabilization are analyzed against critical environmental factors, including pH, temperature, and surfactant concentration. The key findings underscore a growing industrial shift toward sustainable, plant‐derived texturizers that mimic the viscoelastic properties of animal proteins. Ultimately, this review bridges the gap between fundamental macromolecular behavior and practical applications, offering actionable insights for food scientists aiming to innovate clean‐label formulations, optimize structural stability, and enhance the sensory profiles of aerated and gelled matrices.

## Graphical abstract

A comprehensive Graphical Abstract to visualize how different food additives act as gelling and foaming agents. It emphasizes their shared origin as hydrocolloids or proteins but distinct operational mechanisms that transform food materials. The diagram provides a clear path from molecular structure and interaction principles to the specific macro‐scale textures desired in final food applications. Agents: Illustrates common food agents classified by origin (Animal vs. Plant) and their intended function (Gelling vs. Foaming). Mechanism: Simplifies the physical chemistry involved, contrasting the Sol‐to‐Gel Transition (molecular cross‐linking and network formation trapping liquid) with Interfacial Adsorption (lowering surface tension and forming stabilizing films around dispersed gas bubbles). Applications: Connects these mechanisms directly to the resulting food texture types, highlighting Set Textures (jellies, gummies) and Aerated Textures (mousses, foams, beverages).

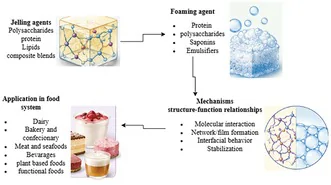

## 1 Introduction

Texture, stability, and sensory quality are among the most important attributes influencing consumer acceptance and market success of food products (Suchánek and Králová 2019; Wang et al. 2023). These characteristics are largely determined by the functional properties of food biopolymers, particularly proteins and polysaccharides, which govern the structural organization of food systems (Asghar et al. 2023). As consumer demand increases for high‐quality, clean‐label, and plant‐based foods, understanding the mechanisms that control food structure and functionality has become increasingly important for food scientists and manufacturers (Inguglia et al. 2023).

Among the various functional properties of food ingredients, gelation and foaming are essential for the development of food texture, appearance, and stability (Deotale et al. 2020; Asghar et al. 2023). Gelation involves the formation of a three‐dimensional network that entraps water and other components, producing products with characteristic firmness, elasticity, and mouthfeel (Razi et al. 2023). Foaming refers to the incorporation and stabilization of gas bubbles within a liquid or semisolid matrix, resulting in light and aerated structures that are important in numerous food products (Aghajanzadeh et al. 2024; Zhao, Liu, et al. 2024; Zhao, Ye, et al. 2024). These functionalities are critical in a wide range of foods, including jams, jellies, confectionery products, dairy desserts, bakery products, mousses, meringues, and whipped toppings (Ochelle et al. 2024; Hu and Meng 2024).

The effectiveness of gelling and foaming systems is influenced by several factors, including the molecular structure of proteins and polysaccharides, pH, temperature, ionic strength, ingredient concentration, and processing conditions (Bhatt et al. 2023; Liu et al. 2023). Conventional ingredients such as gelatin, pectin, albumin, casein, and whey proteins have been extensively used to achieve desirable textural and stability properties in food products (Zhang et al. 2024). However, increasing consumer preference for natural, sustainable, and clean‐label products has encouraged the search for alternative ingredients, particularly plant‐based proteins and polysaccharides capable of delivering comparable functional performance (Hu and Meng 2024; Inguglia et al. 2023).

Despite substantial progress in understanding gelation and foaming phenomena, important challenges remain. Variability in the composition and functionality of natural ingredients can result in inconsistent product quality, while replacing synthetic additives and animal‐derived ingredients without compromising product performance remains difficult (Inguglia et al. 2023; Arab et al. 2023). Furthermore, information regarding the mechanisms, influencing factors, and applications of gelation and foaming is often dispersed across different food systems, limiting the development of a unified understanding of these functionalities.

While several existing reviews have separately addressed the individual mechanisms of hydrocolloid gelation or foam formation, a holistic understanding of their concurrent behaviors in real food systems remains underexplored. This review addresses this critical gap by explicitly focusing on the synergistic cross talk between gelling and foaming functionalities within complex, multi‐phase food matrices, examining how simultaneous interfacial adsorption and continuous‐phase gelation govern macro‐ and Micro‐structural stability. Furthermore, in response to the rapid industrial paradigm shift toward clean‐label and plant‐forward formulations, this work critically evaluates recent advancements in novel plant‐ and algal‐derived hydrocolloids. By systematically comparing their functional trade‐offs and processing resilience against traditional animal‐ or synthetic‐derived stabilizers, this review provides actionable insights for designing sustainable, high performance food structures.

## 2 Literature Search and Review Methodology

This review adopted a systematic approach to collect, evaluate, and synthesize scientific evidence on gelling and foaming agents in food, their mechanisms of action, and applications in food systems (Figure 1). Relevant literature was retrieved from major scientific databases, including Scopus, Web of Science, PubMed, ScienceDirect, and Google Scholar, covering publications up to 2026. A comprehensive search strategy was employed using Boolean operators (“AND” and “OR”) to combine relevant keywords and phrases. Search terms included “gelling agents” AND “food applications,” “foaming agents” AND “food systems,” “hydrocolloids” AND “gel formation,” “protein foams” AND “food products,” “gelation mechanism” OR “food gels,” “foam stability” AND “food processing,” “polysaccharides” AND “gelling properties,” “food texture” AND “hydrocolloids,” and “functional ingredients” AND “food formulation.”

**FIGURE 1:**
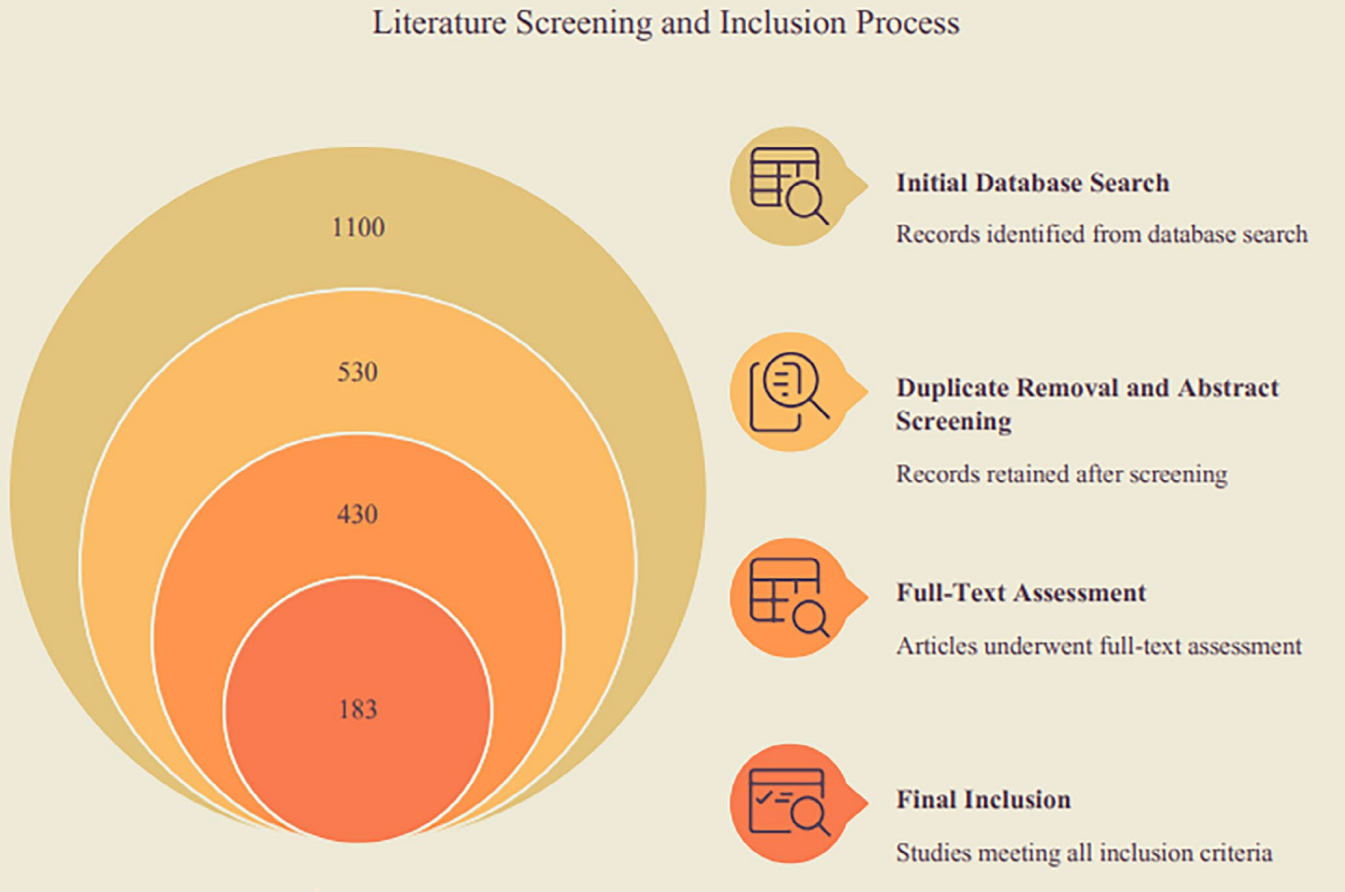
Gelling and Foaming Agents in Food: Mechanism and Applications literature review process.

Studies were selected based on their relevance to the mechanisms, physicochemical properties, functionality, and industrial applications of natural and synthetic gelling and foaming agents. Priority was given to peer‐reviewed journal articles, review papers, book chapters, and authoritative reports published in English. Articles focusing on the structure–function relationships, gelation and foaming mechanisms, interactions with other food components, and emerging trends in food formulation were critically analyzed. The collected literature was then synthesized to provide a comprehensive overview of the current knowledge, technological advances, challenges, and prospects of gelling and foaming agents in the food industry.

A total of 1100 articles were initially identified through database searching. After removing duplicates and conducting preliminary screening, 530 studies were retained based on title and abstract relevance. Subsequently, 430 articles underwent full‐text assessment according to predefined eligibility criteria, resulting in the final inclusion of 183 studies for comprehensive analysis (Table 1).

**TABLE 1:** Summary of literature screening and inclusion process.

Stages	Description	Number of articles
Initial database search	Records identified from Scopus, Web of Science, ScienceDirect, PubMed, and Google Scholar	1100
Duplicate removal and abstract screening	Excluded non‐ relevant and duplicate records	530
Full‐ text assessment	Articles were evaluated for detailed eligibility	430
Final inclusion	Studies meeting all inclusion criteria	183

## 3 Classification of Gelling

### 3.1 Pectin

Pectin, a naturally occurring polysaccharide found in the cell walls of plants, primarily comprises repeating units of α‐(1‐4)‐linked D‐galacturonic acid units (Riseh et al. 2024). Depending on the source plant, the structure of pectin may include homogalacturonan (HG), rhamnogalacturonan I (RG‐I), and rhamnogalacturonan II (RG‐II) domains (Niu et al. 2023). This diversity in composition contributes to the distinct properties exhibited by different types of pectin, such as their ability to form gels, solubility, and rheological behavior. Numerous studies have categorized and explored the gelling properties and mechanisms of pectin, often focusing on factors like its degree of esterification (Said et al. 2023). This section delves into how the presence of esters and other molecules in the pectin network influences its gelation behavior and structural characteristics (Table 2).

**TABLE 2:** The gelling and foaming agents, mechanism, sources, advantages, limitations, and applications used in food products.

Gelling agent	Major mechanism	Source	Advantages	Limitations	Best applications	References
Pectin	Calcium‐mediated crosslinking	Polysaccharides from plants and fruit peels	Plant‐based, clean label	pH dependent	Jams, fruit products	Nath et al. (2022)
Agar	Forms gels through the association of agarose chains into double helices during cooling, which aggregate to create a three‐dimensional network stabilized by hydrogen bonds	Red algae and seaweeds	Strong gel‐forming ability; excellent thermal stability; thermoreversible; effective at low concentrations; suitable for vegetarian and vegan products	Brittle texture; limited elasticity; susceptible to syneresis during storage; performance can be affected by extreme pH conditions	Used as a laxative, vegetarian gelatin substitute, meat and fish items, as well as a texture enhancer in dairy products like yogurt and cheese	Jayappriyan et al. (2021)
Gelatin	Thermoreversible protein gel network	Animal (hydrolytic degradation of collagen protein)	Excellent texture and mouthfeel	Animal origin, heat sensitive	Confectionery, desserts	Alipal et al. (2021)
Egg protein (thermo‐irreversible gels)	Forms gels and stabilizes foams through protein denaturation, unfolding, and aggregation upon heating, creating a permanent three‐dimensional network	Animal (egg)	Excellent gelling and foaming properties; high nutritional value; contributes to desirable texture and structure in bakery and confectionery products	Thermoirreversible gelation; sensitive to overprocessing and pH changes; allergen concerns; animal‐derived source limits use in vegan products	Acts as a gelling agent, imparting the desirable consistency and firmness to a wide array of treats such as custards, puddings, and fruit jellies	Lv et al. (2022)
Alginate	Forms gels through ionic cross‐linking between alginate chains and divalent cations (especially Ca^2+^) via the “egg‐box” model	Polysaccharides derived from seaweed	Strong gel formation under mild conditions; suitable for encapsulation and controlled release; heat‐stable; effective at low concentrations	Requires specific ions for gelation; gels may be brittle; sensitive to calcium concentration and pH; can exhibit syneresis over time	It is the basis of gelling solutions for the production of jellies, gummies, and confectionery items, offering a firm yet chewy texture	Chudasama et al. (2021)
Xanthan gum	Viscosity enhancement rather than true gelation	Polysaccharides produced through fermentation by the bacterium *Xanthomonas campestris*	Stable over wide pH range	Weak gel formation alone	Sauces, beverages	Kassim (2011)
Soy proteins	Stabilizes foams and forms gels through protein unfolding, aggregation, hydrophobic interactions, hydrogen bonding, and disulfide bond formation during heating	Plant (Soybeans)	Plant‐based and sustainable; good emulsifying and foaming properties; high nutritional value; suitable for vegan products	Lower foaming capacity and gel strength than some animal proteins; may impart beany flavor; functionality affected by processing conditions	Serve as gelling agents and texturizers in bakery and confectionery items like cakes, cookies, and candies	Matsumiya and Murray (2016)
Carrageenan	Helix aggregation and ionic interactions	Polysaccharides extracted from red seaweed	Strong gels, dairy compatibility	Consumer concerns in some markets	Dairy systems	Bui (2019)
Whey protein (thermo‐irreversible gels)	Forms gels through heat‐induced denaturation and aggregation, creating networks stabilized by disulfide bonds, hydrophobic interactions, and hydrogen bonding; also stabilizes foams through adsorption at air–water interfaces	A byproduct of cheese production	Excellent gelation, emulsification, and foaming properties; high nutritional quality; forms transparent and elastic gels	Sensitive to pH and ionic strength; heat treatment must be carefully controlled; dairy allergen concerns; unsuitable for vegan applications	Utilized as a gelling agent to modify texture and consistency. It contributes to the creamy mouthfeel and smooth texture of dairy‐based products	Wagner et al. (2020)

High‐methyl (HM) pectins, with a degree of esterification (DE) ranging from 50% and above, predominantly form gels through the cohesion of hydrophobic forces and the formation of hydrogen bonds under specific environmental circumstances (Dias et al. 2024). These conditions typically involve low pH levels (around 2.5–3.5) and the presence of soluble solids such as sucrose (55%–75%) or similar co‐solutes to initiate the gelling process (Said et al. 2023). Sugar plays a crucial role in gel formation by reducing the amount of available water, which stabilizes the junction zones through hydrophobic interactions (Wang et al. 2024). Consequently, the resulting gel features a two‐dimensional (2D) network of interconnected pectin molecules with water and co‐solutes trapped inside, contributing to its ability to resist deformation (Said et al. 2023). The three‐dimensional (3D) network of HM pectin gels is established through junction zones stabilized by hydrogen bonding between carboxyl and secondary alcohol groups, along with hydrophobic interactions involving methyl esters (Kavya et al. 2023). These gels exhibit thermal reversibility, meaning they can undergo gel‐to‐solution transitions with temperature changes (Nasu et al. 2024). When exposed to hot water, HM pectin gels are soluble, and to prevent breaking, they are often used with a dispersion agent like dextrose. Furthermore, pectic polysaccharides, including pectins, are polyelectrolytes whose gelation behavior and interactions with ions are influenced by the solution's ionic strength (Nasu et al. 2024). Elevated ionic strength can alter the pH range for gel formation and augment the formation of robust gel networks by binding divalent cations such as calcium (Ca^2+^), which act as bridges between pectin molecules (Zhao, Khalesi, et al. 2023; Zhao, Zhou, et al. 2023). This phenomenon can also facilitate the establishment of junction zones between pectin molecules, leading to the formation of more resilient gel networks.

Low‐ester pectins have traditionally been the preferred choice for gelling food products, especially in situations where high‐ester pectins may not be as effective in gel formation (Roman‐Benn et al. 2023). Additionally, low‐ester pectins have shown their efficacy in creating stable gels in environments with low to moderate sugar and acidity levels (Cherian et al. 2024). Amidated pectins, a subset of low‐ester pectins, display unique gelling properties, offering a diverse range of functional attributes in the food industry. These distinctive characteristics make them versatile options for a variety of food applications (Roman‐Benn et al. 2023).

### 3.2 Gelatin

Gelatin, thermo reversible gels, is formed through the hydrolytic degradation of collagen protein. Its unique amino acid structure offers several medical benefits (Ahmad et al. 2023). Typically available in tablets, granules, or powders, gelatin can also be dissolved in water before use. Researchers extensively study gelatin as a matrix for three‐dimensional cell culture and as a component of tissue‐engineering scaffolds (Shi et al. 2024). With its high protein content, gelatin is included in certain nutritionally balanced foods and can serve as a substitute for fat and carbohydrates (Floret et al. 2023). Chemically, gelatin comprises 18 varieties of complex amino acids, with approximately 57% consisting of glycine, proline, and hydroxyproline as the major compounds (Ambli et al. 2023). The remaining approximately 43% belong to other distinguished amino acid families such as glutamic acid, alanine, arginine, and aspartic acid. Gelatin consists of 25.2% oxygen, 6.8% hydrogen, 50.5% carbon, and 17% nitrogen (Pereira et al. 2023). It possesses a mixture of single and double unfolded chains with a hydrophilic character. Additionally, the chemical structure of gelatin consists of different polypeptide chains, including α‐chains (individual polymer/single chain), β‐chains (two α‐chains covalently crosslinked), and γ‐chains (three covalently crosslinked α‐chains), with molar masses of approximately 90 × 10^3^, 180 × 10^3^, and 300 × 10^3^ g/mol, respectively (Shamsallah and Rashid 2024).

Gelatin plays a pivotal role in the modern food industry, primarily due to its remarkable gelling properties (Ahmad et al. 2023). In gourmet desserts, gelatin is utilized for texture enhancement, foaming, and achieving clarity (Julie Chandra et al. 2023). It also contributes to stabilizing the structure of various food products. Canned meat items like sausages, frankfurters, loaves, and hams benefit from gelatin by retaining lost juices and facilitating effective heat transfer during cooking. Gelatin finds application in confectionery products such as fruit salads, ice cream, foam, and cottage cheese (Osman and Abo‐Dief 2023). Moreover, owing to its exceptional film‐forming ability and nutritional value, gelatin serves as an edible film and coating material. Additionally, gelatin is employed as a gelling agent in cosmetics and health products, including bath salts, shampoos, sunscreens, body lotions, hair sprays, and facial creams (Ozcan et al. 2023).

### 3.3 Agar

Agar is thermo‐reversible gels which undergo repeated gel–sol transitions upon heating and cooling. It is a hydrocolloid seaweed‐derived polysaccharide with notable gelling properties (Rhein‐Knudsen et al. 2017). Agar is extensively utilized across various industries, with its primary application being in culinary settings, which accounts for 80% of its usage (Padmesh and Singh 2021). Approximately 90% of the extracted agar is employed as a gelling agent in meat and fish items, as well as a texture enhancer in dairy products like yogurt and cheese (Glicksman 2019). Research has explored agar's potential as a binder, particularly in the production of brown rice from corn and red bean flour (Pandya et al. 2022). Agar, being easier to extract compared to other binders like gum or carrageenan, has been investigated for its binding properties (Sousa et al. 2021). When agar‐agar molecules are heated with water to form a gel, they exhibit free movement. In food processing and home cooking, efforts have been made to create medium‐textured mixed gels by combining agar‐agar gel with gelatin jelly (Pandya et al. 2022). These mixed gels are aimed at achieving certain textures that may be more effective than those of individual components alone (Table 2).

Agar also plays a role in reducing syneresis, the phenomenon where water escapes from the gel structure, commonly observed in grass jelly (Boral et al. 2010). Due to its high gel resilience in low pH environments and its ability to bind moisture, agar is favored as a single‐gelling agent in formulations (Pandya et al. 2022). For instance, in green grass jelly drinks, replacing 20% of the original gel with agar can significantly decrease syneresis (Yang et al. 2020). However, careful formulation is required to maintain the desired texture of the drink. Furthermore, agar‐agar, with characteristics akin to carrageenan, can be utilized in the production of edible film (Jayakody et al. 2022). Such edible film, incorporating agar, can serve as packaging material for wrapping jenang cuisine (Pandya et al. 2022). Studies on the characterization of jenang food wrappers made from jackfruit seed flour and agar have shown that the addition of agar affects the film's thickness, tensile strength, elongation, moisture content, and water resistance (Ashila and Rahmatunnisa 2021).

### 3.4 Mechanisms of Gelling Formation

Gelling is the process of forming a gel, which is a semi‐solid structure that provides texture and stability to various food products. The formation of a gel involves intricate molecular interactions that result in the creation of a three‐dimensional network capable of trapping water and other components. The other gel‐forming process is physical and gelation often requires specific conditions such as cooling or heating to trigger the formation of the gel network. Understanding these molecular interactions is crucial for manipulating and optimizing the gelling properties of food items.

#### 3.4.1 Molecular Interactions

##### 3.4.1.1 Hydrogen Bonding

Pectin is a naturally occurring polysaccharide found in the cell walls of fruits. It is widely used in the food industry for its excellent gelling properties through molecular interactions, particularly in jams and jellies (Chandel et al. 2022). The gelation of pectin involves the formation of a network through hydrogen bonding and hydrophobic interactions between its molecules (Feng et al. 2023). Pectin requires specific conditions to gel, typically involving a combination of acid (to lower the pH) and sugar (Zhang et al. 2021). The acidic environment reduces the electrostatic repulsion between negatively charged pectin molecules, allowing them to come closer together (Liang and Luo 2020). Sugar further promotes gelation by binding water molecules, thereby increasing the concentration of pectin and facilitating molecular interactions (Zhao, Liu, et al. 2024; Zhao, Ye, et al. 2024). In jam‐making, fruit pectin is mixed with sugar and heated. When the mixture reaches a certain temperature and pH, the pectin molecules interact through hydrogen bonds, forming a gel that sets upon cooling. The result is a firm, spreadable jam (Abboud et al. 2020).

##### 3.4.1.2 Hydrophobic Interactions

Gelatin is a protein derived from collagen, commonly used in desserts and confectionery for its gel‐forming abilities through molecular interactions (Ahmad et al. 2023). Gelation with gelatin involves the transformation of collagen's triple‐helix structure into individual chains that form a network upon cooling (Lueyot et al. 2021). When gelatin is dissolved in hot water, the heat breaks the hydrogen bonds holding the triple‐helix structure together, resulting in individual gelatin strands (Wanjun et al. 2024). As the solution cools, these strands form new hydrogen bonds and hydrophobic interactions, creating a mesh‐like network that traps water and other solutes, thereby forming a gel (Farokhi et al. 2021). In making gelatin desserts, gelatin powder is dissolved in boiling water and then mixed with cold water or juice (Perera 2021). As the mixture cools in the refrigerator, the gelatin molecules realign and form a semi‐solid gel, giving the dessert its characteristic texture.

##### 3.4.1.3 Double Helix Formation

Agar is a natural polysaccharide obtained from red seaweeds, primarily species of Gelidium and Gracilaria, and is widely recognized for its excellent gelling ability at low concentrations (Pandya et al. 2022). The major structural components of agar are agarose and agaropectin, with agarose being primarily responsible for gel formation. Due to its strong gel strength, transparency, and thermal stability, agar is extensively used in food, pharmaceutical, and microbiological applications (Sousa et al. 2021).

The gelation mechanism of agar is based on a coil‐to‐helix transition of agarose molecules. When agar is heated above its melting temperature (85°C–95°C), the agarose chains exist as randomly dispersed coils in solution. During cooling, these polymer chains undergo conformational changes and align to form ordered double‐helical structures stabilized by intermolecular and intramolecular hydrogen bonds (Tako et al. 2021). The formation of these double helices represents the initial stage of gelation and is responsible for the development of a stable molecular framework.

As cooling continues, the double helices aggregate through additional hydrogen bonding, hydrophobic interactions, and chain entanglements, producing a three‐dimensional network that immobilizes large amounts of water (Sousa et al. 2021). This network formation transforms the liquid solution into a firm gel with high water‐holding capacity and mechanical strength. The unique hysteresis between the gelation temperature (32°C–45°C) and melting temperature (85°C–95°C) gives agar gels exceptional thermal stability compared with many other hydrocolloids, allowing them to remain solid at room temperature and even under moderately elevated temperatures.

Several factors influence the formation and stability of agar double helices. Increasing agar concentration generally enhances gel strength and network density due to the greater availability of agarose chains for helix formation (Padmesh and Singh 2021). The presence of sugars can further strengthen the gel network by promoting polymer–polymer interactions and reducing water mobility. Conversely, extreme acidic conditions may hydrolyze agarose chains, decreasing molecular weight and weakening gel strength. Cooling rate, ionic environment, and molecular weight distribution also affect the extent of helix aggregation and the final textural properties of the gel (Pandya et al. 2022).

The double‐helix aggregation mechanism provides agar gels with several desirable functional properties, including high clarity, rigidity, low syneresis, excellent water retention, and resistance to enzymatic degradation. These properties make agar particularly valuable in food applications such as jellies, confectionery products, puddings, bakery fillings, dairy desserts, and vegan gelatin substitutes. In traditional Asian desserts such as agar‐agar jelly, agar is dissolved in boiling water and mixed with sweeteners and flavoring agents. Upon cooling, the formation of double helices and their subsequent aggregation produces a clear, firm gel that can be molded into various shapes while maintaining structural integrity (De Alwis and Wijesekara 2022).

Beyond conventional food applications, recent research has explored agar‐based composite gels and edible films containing bioactive compounds, nanoparticles, and natural antioxidants. These advanced materials utilize the robust double‐helix network of agar to improve mechanical strength, barrier properties, controlled release behavior, and food preservation performance. Consequently, agar continues to be an important hydrocolloid in the development of sustainable food products and innovative food packaging systems (Sousa et al. 2021).

##### 3.4.1.4 Cross‐Linking by Metal Ions

Carrageenan is a linear sulfated polysaccharide extracted from red seaweeds (Rhodophyceae) and is widely used in the food industry as a gelling, thickening, and stabilizing agent due to its ability to form structured networks through ionic interactions (Jabeen et al. 2025). Its functional properties are mainly derived from the presence of negatively charged sulfate (${‐\mathrm{OSO}}_{3}^{-}$) groups along the polysaccharide backbone, which enable electrostatic interactions with cations such as potassium (K^+^) and calcium (Ca^2+^) (Robal et al. 2017). These metal ions act as cross‐linking agents that connect adjacent carrageenan chains, leading to the formation of a three‐dimensional gel network (Wurm et al. 2020). The gelation process typically involves a coil‐to‐helix transition upon cooling, followed by aggregation of helices stabilized by ionic bridges formed between sulfate groups and metal ions (Lin et al. 2022). The type of carrageenan strongly influences gel behavior: κ‐carrageenan forms strong and brittle gels primarily in the presence of potassium ions, ι‐carrageenan forms softer and more elastic gels in the presence of calcium ions, while λ‐carrageenan does not form true gels but functions mainly as a thickener due to its higher degree of sulfation and weaker ability to form helices (Jabeen et al. 2025).

The nature of the interacting ions also determines the texture and stability of the gel network. Potassium ions fit into the helical cavities of κ‐carrageenan, promoting tight aggregation of double helices and resulting in firm gel structures (Zhao et al. 2025). In contrast, calcium ions, being divalent, form stronger ionic bridges between sulfate groups, enhancing elasticity and water‐holding capacity, particularly in ι‐carrageenan systems (Adfar et al. 2023). These interactions are essential in food systems where texture and stability are important quality attributes. In dairy products such as chocolate milk, carrageenan plays a crucial role in preventing phase separation and improving mouthfeel (Öztürk et al. 2025). Cocoa particles are relatively dense and tend to sediment over time; however, carrageenan forms a weak gel‐like network within the continuous phase that increases viscosity and traps suspended particles, thereby preventing sedimentation (Contreras‐Ramírez et al. 2022). In addition, carrageenan interacts with positively charged regions of casein micelles in milk, further stabilizing the colloidal system through electrostatic attraction and reinforcing the overall network structure.

The performance of carrageenan in food systems is influenced by several factors, including its concentration, the type and concentration of ions present, pH, temperature history, and interactions with proteins (Jabeen et al. 2025). These factors collectively determine gel strength, elasticity, thermal stability, and susceptibility to syneresis. Because of these versatile functional properties, carrageenan is extensively used not only in dairy beverages but also in products such as ice cream, processed meats, and plant‐based milk alternatives. Its ability to create controlled and stable gel networks makes it an important hydrocolloid in modern food formulation and texture engineering.

#### 3.4.2 Physical Processes in Gelling

In addition to molecular interactions, physical processes play a crucial role in the gelling of food products (Aslam et al. 2022). These processes involve changes in temperature, hydration, and mechanical treatment that facilitate the formation of a gel structure. Understanding the physical mechanisms underlying gelation allows food scientists to optimize the texture and stability of various food products.

##### 3.4.2.1 Temperature‐Induced Gelation

Gelatin gelation is a classic example of a temperature‐induced process (Pei et al. 2021). Gelatin is derived from collagen, a protein found in animal connective tissues. Upon heating, the collagen triple helix unwinds into single strands, which then realign into a gel network upon cooling (Tang et al. 2023). When gelatin is dissolved in hot water, the heat breaks the hydrogen bonds that maintain the triple‐helix structure of collagen, resulting in individual gelatin strands (Dille et al. 2021). As the solution cools, these strands re‐form hydrogen bonds and hydrophobic interactions, creating a mesh‐like network that traps water, thereby forming a gel (Liu et al. 2020). In making a gelatin dessert, gelatin powder is first dissolved in boiling water. This heating process denatures the gelatin molecules. The mixture is then allowed to cool, typically in a refrigerator. As the temperature drops, the gelatin molecules reassemble into a three‐dimensional network, creating a firm, semi‐solid gel that holds the dessert's shape (Nishinari 2021).

##### 3.4.2.2 Hydration‐Induced Gelation

Agar, a polysaccharide extracted from red algae, is another example where physical processes like hydration and subsequent cooling lead to gelation (Beaumont et al. 2021). Agar dissolves in boiling water, and as the solution cools, agarose molecules in agar form double helices stabilized by hydrogen bonds (Goncharuk and Dubrovina 2020). These helices aggregate into a three‐dimensional network, entrapping water and other molecules, thereby forming a firm gel. In the preparation of agar‐based jellies, agar is first dissolved in boiling water (Ashila and Rahmatunnisa 2021). Upon cooling, the agar molecules realign into a gel network. This process is highly effective even at relatively low concentrations, allowing for the creation of firm gels used in desserts like “Agar‐Agar Jelly” or scientific applications such as microbial culture media (Ashila and Rahmatunnisa 2021).

##### 3.4.2.3 Ion‐Induced Gelation

Sodium alginate, a polysaccharide derived from brown seaweed, forms gels in the presence of calcium ions (Akbar et al. 2023). This process is widely used in food applications, especially in molecular gastronomy. When a sodium alginate solution is exposed to calcium ions, the ions replace the sodium ions on the alginate chains, allowing the chains to cross‐link and form a gel. This ion exchange is crucial for the formation of the three‐dimensional gel network (Fernando et al. 2020). In the process known as spherification, a liquid containing sodium alginate is dropped into a calcium chloride solution. The calcium ions cause the alginate to gel almost instantly, forming a thin, flexible membrane around the liquid droplet (Xie et al. 2024). This technique is used to create “caviar‐like” spheres filled with various liquids, enhancing both the visual appeal and texture of dishes (Soprani et al. 2021).

##### 3.4.2.4 Pressure‐Induced Gelation

Some hydrocolloids, such as gellan gum, can gel under high pressure, which is particularly useful in food processing (Manzoor et al. 2020). When solutions of gellan gum are subjected to high pressure, the pressure induces the formation of a gel network (Li, He, et al. 2020; Li, Yang, et al. 2020). This process can occur without the need for heating, making it suitable for products that require cold gelation. In the production of certain cold‐set desserts, gellan gum is dissolved in a liquid and then subjected to high pressure (Nishinari 2021). The application of pressure induces the gelation process, resulting in a firm, stable gel that sets without heating, preserving the integrity and freshness of temperature‐sensitive ingredients (Rai et al. 2021).

### 3.5 Factors Affecting Gelling

Gelling, the process of forming a gel‐like structure, is crucial in various food preparations, particularly in jams, jellies, and desserts (Asghar et al. 2023). The formation of a proper gel depends on multiple factors, which can be broadly categorized into the following: the type and concentration of gelling agents, the pH level, the presence of sugars, temperature, and additional ingredients or additives (Cong et al. 2022) (Table 3).

**TABLE 3:** Comparative evaluation of hydrocolloid functionality and structural integrity of gelling under varying conditions (pH, ionic strength, thermal stability, and processing shear).

Hydrocolloid class	Action mechanism	Performance under varying conditions (pH, ionic strength, thermal stability, processing shear)	Advantages	Limitations	Key food applications	References
High Methoxyl Pectin (HMP)	Hydrogen bonding and hydrophobic interactions	pH: Requires acidic pH (2.8–3.5) Ionic Strength: Low ion dependency Thermal Stability: Heat reversible; sensitive to prolonged boiling	Clean flavor release, excellent clarity	Restricted to high‐sugar, low‐pH systems	Jams, jellies, fruit preparations	Xiong et al. (2024)
Low Methoxyl Pectin (LMP)	Ionic cross‐linking via divalent cations	Functions across a wider range (pH 3.0–7.0) Thermally reversible to irreversible depending on calcium level	Works in low‐sugar/dietary products	Excess Ca^2+^ causes syneresis/brittleness	Low‐calorie spreads, dairy gelation	Zhang et al. (2021)
Sodium Alginate	Ionomeric gelation (“egg‐box” model with Ca ^2+^)	pH: Insoluble at < 3.5 (precipitates as alginic acid) Highly sensitive to divalent cations Thermally irreversible gels High shear sensitivity during gelling; shear‐stable post‐setting	Instant cold‐set gelation	Sensitive to calcium sequestration agents	Spherification, restructured foods, bakery fillings	Mohamed et al. (2021)
κ‐Carrageenan	Coil‐to‐helix transition followed by ion‐mediated aggregation	Hydrolyzes at low pH (pH < 4.3) under heating Strongly promoted by K^+^ ions	—	—	—	Li, Munir, et al. (2021) and Li, Wang, et al. (2021)
Xanthan Gum (Foam/Stabilizer)	Disordered random coil creating weak network/high yield stress	Highly stable across broad range (pH 2.0–12.0) Ionic Strength: Stable in high salt	Excellent suspension and foam stabilization	Synergistic increase in viscosity can mask flavor	Sauces, dressings, gluten‐free bakery, aerated foams	Vigata et al. (2021)

### 3.6 Application and Challenges in Food System

The process of gelling has broad and versatile applications (Figure 2) in the food industry, enhancing the texture, stability, and appeal of various products (Yang et al. 2020). Gelatin is commonly used to create gelled desserts, such as gelatin‐based snacks and marshmallows (Ahmad et al. 2023).

**FIGURE 2:**
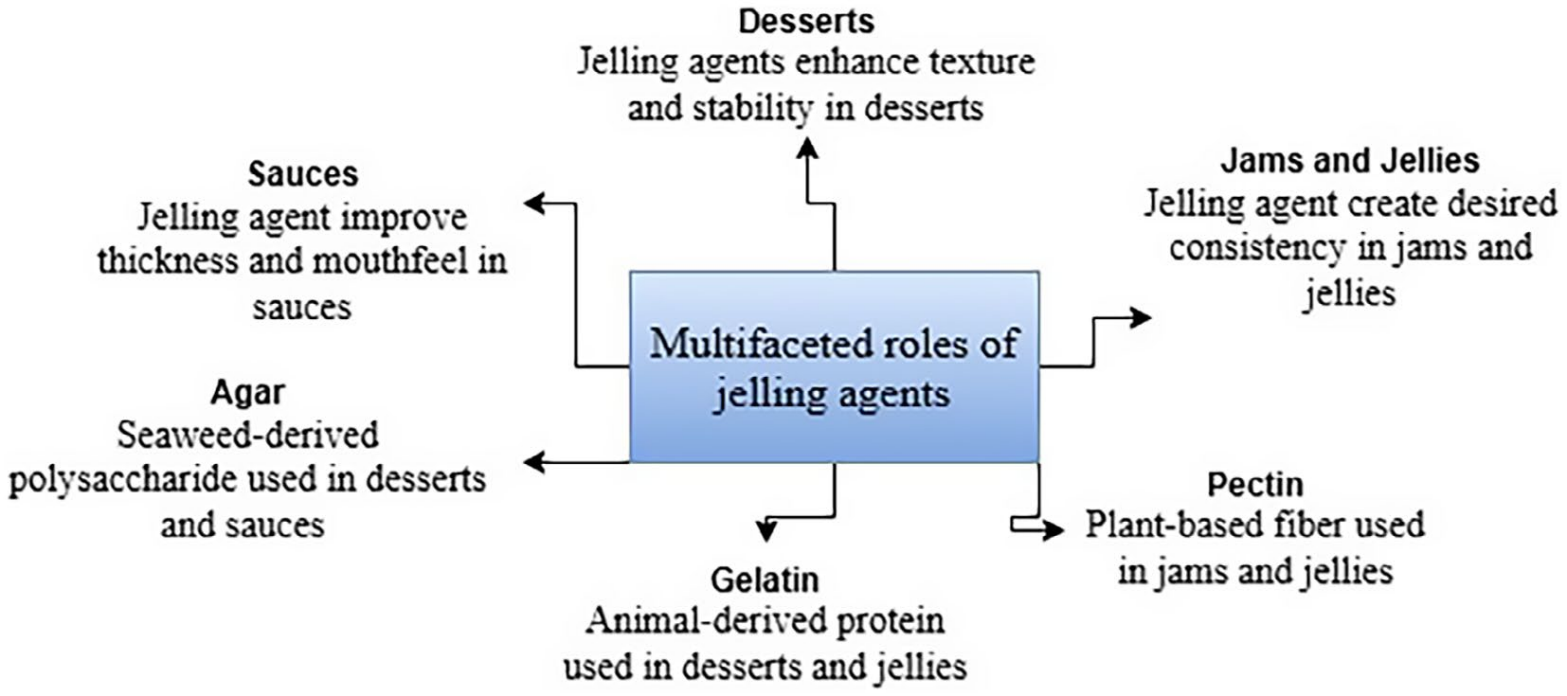
Multifaceted roles of gelling agents.

The gelation process gives these desserts their distinctive texture and structure. Similarly, pectin is the key gelling agent in jams and jellies, providing the desired consistency and spreadability (Toniazzo and Fabi 2023). The gelling process helps to preserve the fruit and create a stable, flavorful product. Gelatin and pectin are used to make gummy candies. The gelation process provides the chewy texture that is characteristic of these sweets. Moreover, carrageenan and gelatin are used in dairy products, particularly in yogurt, cheese, and ice cream (Cheng et al. 2022). In yogurt, gelling agents contribute to texture stabilization, minimize whey separation, and enhance creaminess. Similarly, in processed cheese products, carrageenan and other hydrocolloids are employed to improve textural uniformity, increase product stability, and prevent syneresis (Zhao, Khalesi, et al. 2023; Zhao, Zhou, et al. 2023). Gelling agents such as guar gum and locust bean gum are used in ice cream to improve mouthfeel, reduce ice crystal formation, and enhance stability during storage (Nasrallah et al. 2024). In beverage food products, pectin and other gelling agents are used in fruit juices to enhance mouthfeel and stability (Aggarwal et al. 2020). It helps to maintain a uniform suspension of pulp and other particles. In modern gastronomy, gelling agents like agar and gelatin are used to create cocktail gels, offering a novel way to present beverages with interesting textures and flavors (Precup et al. 2021). In bakeries and pastries, agar and pectin are used in pastry glazes to provide a shiny finish, maintain moisture, and help to create a visually appealing and shelf‐stable product (Albala 2023). Starches and hydrocolloids like gelatin and agar are used in pie fillings, custards, and tarts to provide a thick, stable consistency that does not run or separate when baked or sliced (Gupta et al. 2022).

Despite significant progress in understanding the functional behavior of hydrocolloids and other gelling agents, several challenges still limit their optimal use in food systems (Liu et al. 2024). One of the major issues is the inherent variability of natural sources of gelling agents such as carrageenan, pectin, gelatin, alginate, and starch derivatives (Zang et al. 2025). Because these biopolymers are extracted from biological materials, their chemical composition, molecular weight distribution, degree of substitution, and impurity levels can vary depending on factors such as species, harvest season, geographical origin, and extraction method (Otero et al. 2023). This variability can directly affect functional properties such as gel strength, viscosity, thermal stability, and syneresis behavior, leading to inconsistencies in final product quality. For example, slight differences in the sulfate content of carrageenan or the methoxylation degree of pectin can significantly alter gelation behavior and texture formation in food systems, making standardization difficult at industrial scale.

Another important challenge is the growing consumer demand for clean‐label products, which emphasizes natural, minimally processed ingredients and discourages the use of synthetic additives or chemically modified hydrocolloids (Inguglia et al. 2023). While many gelling agents are naturally derived, industrial processing or chemical modification (such as esterification or oxidation) may lead to consumer skepticism. This has pushed manufacturers to search for “label‐friendly” alternatives that are perceived as natural, safe, and sustainable while still providing the same functional performance in terms of texture, stability, and shelf‐life extension. However, replacing synthetic or modified additives with natural counterparts is not always straightforward, as natural systems often show lower stability under processing stresses such as heat treatment, freezing, or pH changes. As a result, food scientists face the challenge of balancing functionality with consumer expectations, regulatory requirements, and cost‐effectiveness.

In addition to variability and clean‐label concerns, another limitation is the complex interaction of gelling agents with other food components, such as proteins, lipids, sugars, and minerals (Terpou et al. 2025). These interactions can either enhance or inhibit gel formation depending on the system conditions, making formulation design highly complex. For instance, in dairy systems, carrageenan interacts with casein micelles, but excessive ionic strength or inappropriate pH can destabilize the network rather than improve it (Arltoft et al. 2007). Similarly, plant‐based systems often lack the protein structures needed for synergistic gel formation, requiring careful optimization of hydrocolloid blends.

To address these challenges, continuous research and innovation in food science and technology are essential. Advances in extraction and purification techniques are being developed to reduce batch‐to‐batch variability and improve consistency of natural gelling agents. Furthermore, emerging technologies such as enzymatic modification, fermentation‐based production, and nanostructuring of biopolymers are being explored to enhance functionality while maintaining clean‐label status. The use of hydrocolloid blends and synergistic systems is also gaining attention, as combining different biopolymers can compensate for individual limitations and improve overall performance. Overall, overcoming these challenges requires an integrated approach that combines material science, food chemistry, and consumer‐oriented product design.

## 4 Foaming

Foaming in food products is influenced by a complex interplay of chemical, physical, and practical factors (Murray 2020). The nature and concentration of foaming agents, temperature, pH, mechanical agitation, ingredient interactions, equipment, and environmental conditions all play pivotal roles in determining foam formation and stability (Deotale et al. 2020). By comprehensively understanding these factors, food scientists and culinary professionals can optimize foaming processes to achieve desired textures and functionalities, enhancing the sensory appeal and quality of food products.

### 4.1 Mechanisms of Foaming in Food

Foaming is a fascinating and complex phenomenon widely utilized in the food industry to create diverse textures and sensory experiences (Fameau 2021). Central to the process of foaming are proteins and surfactants, which play critical roles in reducing surface tension and stabilizing bubbles (Petkova et al. 2020). Understanding these mechanisms from various food industry perspectives: culinary, dairy, bakery, and beverage provides insights into how different products achieve their unique textures and qualities.

#### 4.1.1 Protein and Surfactant Roles

Proteins are natural foaming agents that significantly reduce surface tension, allowing the formation of stable air bubbles (Masoule et al. 2023). In culinary applications, proteins from egg whites and dairy are predominant (Lima et al. 2022). When egg whites are beaten, the proteins unfold and realign at the air‐water interface, forming a thin film that encapsulates air bubbles (Chi and Ma 2023). This action not only reduces the surface tension but also provides structural support, creating a stable foam that is essential for dishes like meringues and soufflés. Dairy proteins, such as casein and whey, function similarly in products like whipped cream and mousses, where their amphiphilic nature (having both hydrophilic and hydrophobic regions) helps to stabilize the air bubbles within the liquid matrix (Xiong et al. 2020).

Surfactants, on the other hand, are molecules with a distinct ability to reduce surface tension more efficiently than proteins (Fatma et al. 2021). In the bakery and confectionery sectors, surfactants such as lecithin and monoglycerides are commonly used (Sharma and Sharma 2021). Lecithin, derived from soybeans or egg yolks, is a phospholipid that aligns at the air‐water interface, reducing surface tension and forming a stable foam (Shchipunov 2020). In bakery products, this property is crucial for creating light, airy textures in cakes and pastries. Monoglycerides, used in whipped toppings and ice creams, provide similar benefits by stabilizing air bubbles and improving the overall texture and mouthfeel of the product (Xie et al. 2023).

#### 4.1.2 Bubble Stabilization

Once bubbles are formed, their stabilization is paramount to maintaining the desired texture and structure of the food product (Zhu et al. 2024). The stability of the foam is maintained by the formation of a protective layer around the gas bubbles, which prevents them from coalescing and collapsing (Langevin and Langevin 2020). In dairy products like whipped cream and ice cream, the stabilization of bubbles is achieved through the interplay of fat globules and proteins (Ho et al. 2020). The fat globules partially coalesce, forming a network that traps and stabilizes air bubbles (Liu et al. 2022). This network is further reinforced by proteins that create a secondary layer around the bubbles, ensuring long‐lasting stability (Briceño‐Ahumada et al. 2022). The result is a smooth, creamy texture that is essential for consumer satisfaction. In beverages, especially carbonated drinks and specialty coffees, foam stability is equally important (Deotale et al. 2020). In beer and sparkling wines, proteins and other surface‐active compounds from the brewing ingredients contribute to foam stability (Vilela et al. 2020). These compounds form a resilient layer around the bubbles, creating the characteristic head that enhances the visual appeal and sensory experience of the beverage. In specialty coffees like cappuccinos, the foaming of milk is achieved through the denaturation and stabilization of milk proteins, primarily casein and whey (S. Deotale et al. 2020). The microfoam created by steaming milk is stabilized by these proteins, providing a rich and creamy texture that complements the coffee (Zakidou et al. 2022). In the bakery sector, foams are crucial for products like angel food cakes and chiffon cakes (Li, Huang, et al. 2022; Li, Wang, et al. 2022). Egg whites are beaten to incorporate air, and the proteins stabilize the bubbles to create a light, airy batter. The foam stability is further enhanced by adding stabilizers such as cream of tartar, which helps maintain the structure during baking (Thaliath 2024). The result is a cake with a tender crumb and light texture that is highly prized.

### 4.2 Foaming Agents in Food

Foaming agents are essential additives in the food industry, playing a critical role in creating the unique textures and consistencies that enhance the sensory experience of various culinary products (Xu et al. 2020). These agents facilitate the formation of foam, which is a colloidal system consisting of gas bubbles dispersed in a liquid or solid matrix. Foams are integral to the appeal and functionality of numerous food items, from the lightness of meringues and soufflés to the creamy consistency of whipped toppings and mousse (Deotale et al. 2023). This review will explore some of the most common foaming agents used in food production, highlighting their sources, applications, and properties (Table 4).

**TABLE 4:** The foaming agent, source, and its application.

Foaming agent	Source	Application in the food industry	References
Egg white protein	Protein based	It boasts exceptional stabilizing properties in the traditional preparation of numerous foamed products such as delicate meringues, velvety mousses, and a myriad of baked goods	Razi et al. (2023)
Soy lecithin	A mixture of phospholipids, glycolipids, neutral lipids, and carbohydrates	It is useful in creating foams in a variety of culinary applications, including sauces, dressings, and emulsions	Ketomäki et al. (2021)
Gelatin	Protein‐based (derived from collagen found in animal bones and connective tissues)	Gelatin is used in the production of gummy candies and marshmallows, where it acts as a foaming agent to create the desired texture and mouthfeel	Ahmad et al. (2023)
Methylcellulose	Cellulose derivative	Used in food processing to improve the texture and stability of products such as ice cream, whipped toppings, and dairy alternatives	Grover (2020)
Sucrose esters	Esterification of sucrose with fatty acids	In carbonated beverages, sucrose esters are added to improve foam stability and reduce foam collapse, enhancing the visual appeal of the product	Liu and Binks (2021)
Glycerol Monostearate (GMS)	Glycerol and stearic acid	In carbonated beverages and whipped toppings, GMS improves foam stability and prevents collapse, enhancing the visual appeal and overall quality of the product	Habibi (2022)
Sodium Lauryl Sulfate (SLS)	Lauric acid found in coconut oil	Used as an indirect food additive, such as in cleaning and sanitizing food processing equipment	Bondi et al. (2020)

#### 4.2.1 Egg White

Egg whites are perhaps the most traditional and widely recognized foaming agent in the culinary world (Razi et al. 2023). Derived from chicken eggs, egg whites are rich in proteins like ovalbumin and ovotransferrin, which are responsible for their excellent foaming capacity (Li, Huang, et al. 2022; Li, Wang, et al. 2022). When beaten, these proteins unfold and trap air, creating a stable foam. The addition of acids, such as cream of tartar, can further enhance foam stability. Egg whites are indispensable in making meringues, mousses, and soufflés, where their ability to form and maintain a stable foam is crucial (Shidara et al. 2023).

#### 4.2.2 Soy Lecithin

Soy lecithin, sourced from soybeans, serves as both a foaming agent and an emulsifier (Farid et al. 2023). It is particularly useful in creating foams in a variety of culinary applications, including sauces, dressings, and emulsions. Lecithin works by lowering the surface tension of liquids, which promotes the formation of foam (Tabaniag et al. 2023). Its versatility makes it a popular choice for both hot and cold applications, adding a light, airy texture to foods without altering their flavor profile (Hu and Meng 2024).

#### 4.2.3 Gelatin

Gelatin, derived from animal collagen found in bones and connective tissues, is a widely used foaming agent in the food industry (Ahmad et al. 2023). It is essential in the production of marshmallows, gummy candies, and various desserts. Gelatin provides both foam stability and a desirable texture, melting at body temperature to offer a pleasant mouthfeel (Siburian et al. 2020). Its ability to form a gel when cooled also makes it valuable in creating stable foams that maintain their structure over time (Abdollahi et al. 2020).

#### 4.2.4 Methylcellulose

Methylcellulose, a chemically modified form of cellulose, is known for its unique property of forming gels when heated, making it ideal for hot foams (Grover 2020). This agent is used in sauces, foams, and gluten‐free baking, where its reversible gelling properties add significant versatility (Hu and Meng 2024). Methylcellulose can create foams that remain stable even at high temperatures, providing innovative textures and mouthfeel in hot culinary applications (Jarpa‐Parra and Chen 2021).

#### 4.2.5 Sucrose Esters

Sucrose esters, produced from sucrose and fatty acids, are effective foaming agents in products like ice creams, cakes, and whipped toppings (Liu and Binks 2021). It works by reducing the surface tension of water, which aids in foam formation and stabilization (Sardar et al. 2024). Sucrose esters help maintain the integrity of air cells in foams, extending their shelf life and improving texture (Chen et al. 2024). It can be used to modify the texture of food products such as sauces, gravies, and fillings, providing a smoother and more consistent mouthfeel (Liu and Binks 2021).

#### 4.2.6 Glycerol Monostearate (GMS)

Glycerol Monostearate, derived from glycerol and stearic acid, is commonly used in baked goods, whipped cream, and ice cream (Malvano et al. 2024). It acts as a stabilizer and emulsifier, enhancing the volume and texture of foams (Andriotis et al. 2022). GMS helps to maintain the consistency of foam, ensuring that products remain light and airy (Mehta and Pinto 2023). It can be used to modify the texture of food products such as sauces, gravies, and fillings, providing a smoother and more consistent mouthfeel. It acts as an emulsifier in food products like dressings, sauces, and mayonnaise, improving stability and preventing oil–water separation (Tan and McClements 2021).

#### 4.2.7 Sodium Lauryl Sulfate (SLS)

Sodium lauryl sulfate, a surfactant obtained from lauric acid found in coconut oil, is a potent foaming agent used in beverages, bakery products, and whipped toppings (Guo et al. 2022). It is highly effective at creating foams due to its ability to reduce surface tension significantly (Sahu et al. 2021). However, SLS is used in very low concentrations to prevent any potential adverse effects on taste or safety (Nirmala et al. 2021).

#### 4.2.8 Monoglycerides and Diglycerides

Monoglycerides and diglycerides, derived from glycerol and fatty acids, are prevalent in dairy products, baked goods, and margarine (Ferreira et al. 2021). These agents aid in aeration and foam stabilization, often used in combination with other emulsifiers to enhance performance (Wang et al. 2021). They contribute to the smooth, consistent texture of foams in various food products (Ho et al. 2020).

#### 4.2.9 Xanthan Gum

Xanthan gum, produced through the fermentation of glucose or sucrose by *Xanthomonas campestris* bacteria, is utilized in salad dressings, sauces, and gluten‐free products (Samrot et al. 2023). It adds viscosity and stability to foams, and when used with other gelling agents like locust bean gum, it can form gels that enhance foam structure (Briceño‐Ahumada et al. 2022).

#### 4.2.10 Caseinate

Caseinate, derived from milk proteins, is an excellent foaming agent used in protein shakes, coffee creamers, and processed cheeses (Hammam et al. 2021). The amphiphilic nature of casein proteins provides exceptional foaming properties, resulting in stable and smooth‐textured foams (Liang and Luo 2020).

#### 4.2.11 Emerging Plant‐Based Foaming Agents

In addition to conventional foaming agents such as egg and dairy proteins, considerable attention has recently been directed toward plant‐based alternatives (Cenaque et al. 2026). Among these, aquafaba, the cooking water derived from chickpeas and other legumes, has emerged as a promising natural foaming agent (Koriyama et al. 2025). Aquafaba contains proteins, polysaccharides, saponins, and other soluble compounds that contribute to its ability to form and stabilize foams (Koriyama et al. 2025). Studies have demonstrated that aquafaba can provide foaming capacity and stability comparable to egg whites in certain food applications, making it an attractive ingredient for vegan, allergen‐free, and clean‐label formulations (Buhl et al. 2019). The growing interest in aquafaba reflects the broader trend toward sustainable and plant‐based ingredients capable of replacing traditional animal‐derived foaming agents while maintaining desirable functional properties.

### 4.3 Factors Affecting Foaming

Foaming is a complex process influenced by a multitude of factors that can significantly alter the formation, stability, and properties of foam in food products (Hjelt et al. 2022). Understanding these factors from different perspectives: chemical, physical, and practical is essential for optimizing foaming processes in culinary and food science applications (Murray 2020). This comprehensive analysis delves into the key factors affecting foaming, examining their impact and interrelation.

#### 4.3.1 Chemical Perspective

From a chemical standpoint, the nature and concentration of foaming agents play a crucial role in foam formation and stability (Zhan et al. 2022). Proteins, such as egg whites and casein, are amphiphilic molecules that can reduce surface tension and form a flexible film around air bubbles, stabilizing the foam (Han et al. 2023). The protein concentration must be optimized, as too little may result in weak foams, while too much can lead to overly stiff and unstable foams (Li, He, et al. 2020; Li, Yang, et al. 2020). Surfactants are another category of foaming agents that reduce surface tension more effectively than proteins (Krzan et al. 2024). Their molecular structure allows them to align at the air‐water interface, forming a barrier that prevents the coalescence of air bubbles (Nguyen et al. 2023). The hydrophilic–lipophilic balance (HLB) of surfactants is a critical factor; an appropriate HLB value ensures optimal foam stability and consistency (Sarkar et al. 2021).

#### 4.3.2 Physical Perspective

Physical factors such as temperature, pH, and mechanical agitation significantly impact foaming (Deotale et al. 2020). Temperature affects the solubility and flexibility of foaming agents. For instance, proteins like egg whites are more effective at room temperature, where they can unfold and form stable foams (Ding et al. 2022). However, excessive heat can denature proteins, leading to foam collapse (Razi et al. 2023). Similarly, the temperature must be controlled to maintain the integrity of surfactant‐based foams. The pH level of the foaming medium can also influence foam properties. Proteins have an isoelectric point (pI) at which they carry no net charge, leading to minimal solubility and reduced foaming ability (Figueroa‐González et al. 2022). Adjusting the pH away from the pI enhances protein solubility and foaming capacity. For example, adding an acid‐like cream of tartar to egg whites lowers the pH, improving foam stability (Wang et al. 2022).

Mechanical agitation, including whipping, shaking, or the use of mechanical frothers, introduces air into the liquid, creating bubbles (Xiong et al. 2023). The intensity and duration of agitation affect the size and distribution of bubbles, with vigorous agitation producing finer, more stable foams. However, over‐agitation can break down the foam structure, leading to collapse (Li, Munir, et al. 2021; Li, Wang, et al. 2021).

#### 4.3.3 Practical Perspective

In practical applications, ingredient interactions and environmental conditions are critical to foaming success. Ingredients such as fats and oils can inhibit foam formation by disrupting the film around air bubbles (Ho et al. 2020). Therefore, foaming agents must be carefully selected and combined to ensure compatibility and desired functionality. For instance, in bakery products, the presence of fat can interfere with egg white foams, necessitating adjustments in ingredient proportions and processing techniques (Aghajanzadeh et al. 2024). The equipment and techniques used in food preparation also influence foaming. High‐shear mixers and siphons are often employed to produce consistent and stable foams (Mathijssen et al. 2023). The choice of equipment can affect the texture and stability of the final product. In molecular gastronomy, tools like nitrous oxide siphons are used to create innovative foams with unique textures and flavors, highlighting the importance of technology in modern culinary practices (Precup et al. 2021). Environmental factors such as humidity and air pressure can also impact foam stability. High humidity can cause foams to become wetter and less stable, while variations in air pressure can affect bubble formation and size (Tapia‐Blácido et al. 2022). Understanding these external conditions is essential for maintaining consistent foam quality, particularly in commercial food production.

### 4.4 Application and Challenges and Foaming Capacity in Different Aspects of Food Preparations

Foaming capacity is a crucial characteristic in the food science world, defining the ability of a substance to incorporate and stabilize gas bubbles within a liquid or semi‐solid matrix (Deotale et al. 2020). This property is instrumental in achieving the light and airy textures that are essential in many food products, ranging from delicate pastries to robust dairy products. Understanding and manipulating foaming capacity can significantly impact the quality, texture, and consumer appeal of various foods.

In the realm of baked goods, foaming capacity is central to the creation of light and airy textures (Csurka et al. 2022). Products such as angel food cakes, soufflés, and meringues rely heavily on the foaming ability of egg whites (Li, Huang, et al. 2022; Li, Wang, et al. 2022). When beaten, egg whites form a stable foam that traps air, expanding during baking to produce a light, fluffy structure (Alavi et al. 2020). The stability of this foam is critical; any compromise can result in a dense, unappealing product. Ingredients like cream of tartar are often added to enhance foam stability, ensuring consistent quality and texture (Fakhernia et al. 2023). Foaming capacity also plays a vital role in the dairy industry, particularly in the production of whipped cream, mousses, and certain types of cheeses (Briceño‐Ahumada et al. 2022). For instance, whipped cream relies on the ability of cream to form a stable foam when air is incorporated. The presence of fat globules in the cream helps stabilize the foam, giving it a smooth, creamy texture (Waldron et al. 2020). In cheeses like ricotta and cottage cheese, the ability to maintain a light, airy texture can enhance the overall mouthfeel and consumer satisfaction (Potter and Vargo 2023).

In beverages, foaming capacity is essential for products like beer, sparkling wines, and specialty coffees (Deotale et al. 2020). The foam, or “head” on beer, is a result of the foaming properties of proteins and other compounds present in the brew (Bamforth 2023). This foam not only affects the visual appeal but also impacts the aroma and flavor experience. In sparkling wines, the tiny bubbles that rise to the surface create a sensory experience that is highly valued by consumers (Liger‐Belair and Cilindre 2021). Specialty coffees, such as cappuccinos and lattes, depend on the frothing ability of milk to create a rich, creamy foam that complements the coffee's flavor (Angeloni et al. 2021). In confectionery, the foaming capacity is crucial for products like marshmallows, nougats, and whipped candies (Gunes et al. 2022). Gelatin is often used as a foaming agent in these products, providing both foam stability and a pleasant texture (Alipal et al. 2021). The ability of gelatin to trap air and maintain a stable foam during the cooling process is essential for achieving the desired lightness and chewiness in these sweets (Briceño‐Ahumada et al. 2022). Without adequate foaming capacity, these confections would lack their characteristic texture and appeal.

Foaming capacity is also significant in various processed foods, such as aerated chocolate bars, instant whipped toppings, and certain snack foods (Pugh et al. 2023). In aerated chocolates, the incorporation of gas bubbles creates a lighter texture, enhancing the eating experience. Ingredients like soy lecithin or milk proteins are often employed to ensure stable foam formation and retention (Amagliani et al. 2021). Instant whipped toppings use emulsifiers and stabilizers to mimic the foaming properties of traditional cream, providing convenience without sacrificing texture or stability (Lapčíková et al. 2024). In the preparation of sauces and dressings, foaming capacity can influence texture and mouthfeel. For example, sauces that are aerated to incorporate air can provide a lighter, more delicate texture, enhancing the dish's overall presentation and sensory appeal. Emulsifiers like lecithin and stabilizers such as xanthan gum are often used to create stable foams that hold their structure over time, even in acidic or complex flavor environments (Hu and Meng 2024).

For gluten‐free baking, where traditional wheat flour's gluten network is absent, the foaming capacity becomes a critical factor in achieving desirable textures (Gómez 2022). Ingredients such as methylcellulose and egg whites are often utilized to provide the necessary structure and aeration (Briceño‐Ahumada et al. 2022). These agents help to trap air within the batter, allowing gluten‐free baked goods to rise and maintain a light, fluffy texture similar to their gluten‐containing counterparts. Modern gastronomy often leverages foaming capacity to create innovative dishes and textures (Hu and Meng 2024). Techniques such as using siphons to create foams and airs from liquids and purees have become popular in avant‐garde cooking. Soy lecithin, for example, is frequently used in molecular gastronomy to create stable, airy foams that can be flavored with a wide range of ingredients, adding both visual and textural interest to a dish (Kozakiewicz and Cossuta 2021).

While foaming capacity offers numerous benefits, it also presents challenges. Maintaining foam stability can be difficult, as factors such as pH, temperature, and the presence of fats or alcohol can affect foam formation and longevity (Deotale et al. 2020). Moreover, the sensory properties of foaming agents must be carefully balanced to avoid off‐flavors or undesirable textures (Best 2023). Food scientists and chefs must consider these factors when selecting and using foaming agents to ensure the final product meets consumer expectations.

## 5 Conclusion

This review highlights the fundamental mechanisms of gelation and foaming, two essential functionalities in food systems that rely on the formation and stabilization of structured networks. Although gels and foams differ in their physical architecture, both are governed by complex interactions among biopolymers and are strongly influenced by environmental factors such as pH, temperature, ionic strength, and molecular structure. Traditional gelling and foaming agents, including pectin, gelatin, albumin, and casein, continue to play important roles in food formulation. However, increasing demand for sustainable, clean‐label, and plant‐based products has exposed limitations associated with animal‐derived ingredients and synthetic stabilizers. Consequently, there is growing interest in alternative biopolymers derived from plant proteins, legumes, and agricultural by‐products such as aquafaba. Future research should focus on improving the functionality of plant‐based ingredients through a deeper understanding of their interfacial behavior, adsorption kinetics, and rheological properties. Advanced processing technologies, including high‐intensity ultrasound, cold plasma, and high‐pressure processing, offer promising approaches for tailoring the structural and functional properties of non‐animal biopolymers to mimic conventional gelling and foaming agents. In addition, the development of polysaccharide–protein hybrid systems may provide enhanced stability, resistance to thermal degradation, and reduced syneresis in clean‐label food formulations. Overall, bridging fundamental knowledge of macromolecular interactions with industrial‐scale applications will be essential for developing the next generation of stable, sustainable, and sensory‐optimized food products. Such advancements will support innovation in food design while meeting evolving consumer and environmental demands.

## Author Contributions

**Messenbet Geremew Kassa:** writing – review and editing, writing – original draft. **Desye Alemu Teferi:** writing – review and editing. **Neela Satheesh:** writing – review and editing. **Aynadis Molla Asemu:** investigation, writing – review and editing.

## Funding

The authors have nothing to report.

## Conflicts of Interest

The authors declare no conflicts of interest.

## Data Availability

The data that support this review are available from the corresponding author upon reasonable request.
